# National Preclinical Sepsis Platform: developing a framework for accelerating innovation in Canadian sepsis research

**DOI:** 10.1186/s40635-020-00366-4

**Published:** 2021-03-19

**Authors:** Asher A. Mendelson, Casey Lansdell, Alison E. Fox-Robichaud, Patricia Liaw, Jaskirat Arora, Jean-François Cailhier, Gediminas Cepinskas, Emmanuel Charbonney, Claudia dos Santos, Dhruva Dwivedi, Christopher G. Ellis, Dean Fergusson, Kirsten Fiest, Sean E. Gill, Kathryn Hendrick, Victoria T. Hunniford, Paulina M. Kowalewska, Karla Krewulak, Christian Lehmann, Kimberly Macala, John C. Marshall, Laura Mawdsley, Braedon McDonald, Ellen McDonald, Sarah K. Medeiros, Valdirene S. Muniz, Marcin Osuchowski, Justin Presseau, Neha Sharma, Sahar Sohrabipour, Janet Sunohara-Neilson, Gloria Vázquez-Grande, Ruud A. W. Veldhuizen, Donald Welsh, Brent W. Winston, Ryan Zarychanski, Haibo Zhang, Juan Zhou, Manoj M. Lalu, Sepsis Canada’s National Preclinical Sepsis Platform

**Affiliations:** 1 Department of Medical Biophysics, Schulich School of Medicine and Dentistry, University of Western Ontario, London, ON Canada; 2 Centre for Critical Illness Research, Lawson Health Research Institute, London, ON Canada; 3 Regenerative Medicine Program, Ottawa Hospital Research Institute, Ottawa, ON Canada; 4 Department of Medicine, McMaster University, Hamilton, ON Canada; 5 Thrombosis and Atherosclerosis Research Institute, Hamilton, ON Canada; 6 Department of Medical Sciences, McMaster University, Hamilton, ON Canada; 7 Centre de Recherche du Centre Hospitalier de l’Université de Montréal, Montreal, QC Canada; 8 Département de Médecine, Université de Montréal, Montreal, QC Canada; 9 Keenan Research Centre for Biomedical Science, Unity Health Toronto, Toronto, ON Canada; 10 Interdepartmental Division of Critical Care Medicine, University of Toronto, Toronto, ON Canada; 11 Department of Medicine, University of Toronto, Toronto, ON Canada; 12 Robarts Research Institute, University of Western Ontario, London, ON Canada; 13 Clinical Epidemiology Program, Ottawa Hospital Research Institute, Ottawa, ON Canada; 14 Department of Critical Care Medicine, Cumming School of Medicine, University of Calgary, Calgary, AB Canada; 15 Department of Medicine, Schulich School of Medicine and Dentistry, University of Western Ontario, London, ON Canada; 16 Department of Communications, Global Sepsis Alliance, Canada Sector, Toronto, ON Canada; 17 Department of Anesthesia, Pain Management and Perioperative Medicine, Dalhousie University, Halifax, NS Canada; 18 Department of Critical Care Medicine, Royal Alexandra Hospital, University of Alberta, Edmonton, AB Canada; 19 Department of Surgery, University of Toronto, Toronto, ON Canada; 20 Snyder Institute for Chronic Diseases, Cumming School of Medicine, University of Calgary, Calgary, AB Canada; 21 Ludwig Boltzmann Institute for Experimental and Clinical Traumatology, Vienna, Austria; 22 School of Epidemiology and Public Health, University of Ottawa, Ottawa, ON Canada; 23 Animal Resources Centre, University Health Network, Toronto, ON Canada; 24 Department of Internal Medicine, Section of Critical Care, University of Manitoba, Winnipeg, MB Canada; 25 Department of Medical Microbiology and Infectious Diseases, University of Manitoba, Winnipeg, MB Canada; 26 Department of Medicine, University of Calgary, Calgary, AB Canada; 27 Department of Internal Medicine, Section of Hematology/Medical Oncology, University of Manitoba, Winnipeg, MB Canada; 28 Interdepartmental Division of Critical Care Medicine, Department of Anesthesia, University of Toronto, Toronto, ON Canada; 29 Department of Anesthesiology and Pain Medicine, The Ottawa Hospital, 501 Smyth Road, PO Box 201B, Ottawa, ON K1H 8L6 Canada; 30 Department of Physiology and Pharmacology, Schulich School of Medicine and Dentistry, University of Western Ontario, London, ON Canada; 31 Department of Critical Care Medicine, University of Toronto, Toronto, ON Canada; 32 Department of Biochemistry and Molecular Biology, University of Calgary, Calgary, AB Canada; 33 Interdepartmental Division of Critical Care Medicine, Department of Physiology, University of Toronto, Toronto, ON Canada

**Keywords:** Sepsis, Experimental models of sepsis, Multicentre preclinical, Translation, Reproducibility, Multi-stakeholder, Integrated knowledge translation, PIRO

## Abstract

Despite decades of preclinical research, no experimentally derived therapies for sepsis have been successfully adopted into routine clinical practice. Factors that contribute to this crisis of translation include poor representation by preclinical models of the complex human condition of sepsis, bias in preclinical studies, as well as limitations of single-laboratory methodology. To overcome some of these shortcomings, multicentre preclinical studies—defined as a research experiment conducted in two or more research laboratories with a common protocol and analysis—are expected to maximize transparency, improve reproducibility, and enhance generalizability. The ultimate objective is to increase the efficiency and efficacy of bench-to-bedside translation for preclinical sepsis research and improve outcomes for patients with life-threatening infection. To this end, we organized the first meeting of the National Preclinical Sepsis Platform (NPSP). This multicentre preclinical  research collaboration of Canadian sepsis researchers and stakeholders was established to study the pathophysiology of sepsis and accelerate movement of promising therapeutics into early phase clinical trials. Integrated knowledge translation and shared decision-making were emphasized to ensure the goals of the platform align with clinical researchers and patient partners. 29 participants from 10 independent labs attended and discussed four main topics: (1) objectives of the platform; (2) animal models of sepsis; (3) multicentre methodology and (4) outcomes for evaluation. A PIRO model (predisposition, insult, response, organ dysfunction) for experimental design was proposed to strengthen linkages with interdisciplinary researchers and key stakeholders. This platform represents an important resource for maximizing translational impact of preclinical sepsis research.

## Background and rationale

Sepsis is the life-threatening organ dysfunction caused by a dysregulated host response to infection [1] and accounts for one-fifth of all deaths worldwide [2]. In 2017, the World Health Organization adopted a resolution to improve the prevention, diagnosis and clinical management of sepsis [3]. Preclinical sepsis research—defined here as laboratory-based animal and basic science research—plays an essential role in this overall strategy, and has contributed substantially to our understanding of sepsis pathobiology and organ dysfunction [4, 5]. However, despite decades of preclinical research findings, there remains no experimentally derived therapies for sepsis successfully adopted into routine clinical practice [6].

Several factors contribute to impediments in translation to the bedside as described in Table 1. Clinical sepsis is highly heterogenous, and imperfectly reflected in a single animal model. These animal models are frequently criticized for inadequately representing and over-simplifying the sepsis syndrome. For example, research animals are often genetically identical, and almost exclusively healthy (no pre-morbid conditions, co-interventions, medications and/or environmental stressors). Moreover, historically, significant sex bias has led to under-representation of female animals in sepsis experiments, and although recent efforts have been made to address these disparities, sex-based analysis continues to be underreported [7]. In addition, sepsis is often mimicked with non-bacterial surrogates (e.g. endotoxin) that may reduce validity of findings. Conversely, when true models of infection are used polymicrobial abdominal sepsis is the prevailing locus of infection, which may reduce generalizability to other forms of sepsis. There is also a disconnect from clinical management as standard therapies for sepsis (e.g. antibiotics, intravenous fluids, mechanical ventilation) are often omitted in experimental models. Similarly, novel therapeutics are often tested in these animal models as a pre- or co-treatment with septic inoculation, a timing of intervention that has little clinical correlation. Practically, it is often difficult to maintain septic animals for prolonged periods of time, to successfully institute life support over days, and to follow the natural history of sepsis survival and recovery (weeks to months). Ethical considerations for humane animal care limit direct observations of organ failure and mortality, and preclinical endpoints do not always function as appropriate surrogates for clinical outcomes. Finally, consideration for variability in host response and outcome (even in genetically identical animals) is rarely accounted for, leading to inadequately powered studies using small groups of animals.

**Table 1:** Potential factors contributing to the lack of “bench-to-bedside” translational success of preclinical sepsis research

Domain	Preclinical model	Human condition
Construct validity		
Patient population	Historically male, however more female animals in recent years Healthy Young/juvenile Limited environmental exposure Genetically homogeneous	Female and male Medical comorbidities Old and young Environmental stressors Genetically diverse
Site of infection	Non-bacterial surrogates (e.g. endotoxin) Polymicrobial abdominal/enteric Gram-negative Pneumonia (rare) Fungi/protozoa (rare) Virus (very rare)	Soft tissue Gram-positive Abdominal Gram-negative, including biliary Pneumonia (common) Virus (common) Fungi/protozoa
Intercurrent therapy	None Antibiotics (monotherapy) Fluids Anesthesia/analgesia Experimental therapy	Antibiotics (poly-therapy) Fluids Blood products Vasopressors/Inotropes Sedation/analgesia Baseline medication regimen Adjunct therapies (e.g. steroids, heparin)
Outcomes	Non-mortality surrogate Short term Organ failure (often single) Molecular biomarkers (common) Organ histology	Mortality Short and long term ICU/hospital length of stay Validated multi-organ failure score Molecular biomarkers (rare) Organ histology (very rare)
Research methodology		
Biostatistics	Lack of sample size calculation	Study powered to detect difference in pre-specified outcome
Reduce bias	Randomization rare Lack of blinding	Randomization Double-blinded
Standardization	Single centre Variations in practice	Multicentre Shared protocol
Reporting	Inconsistent Incomplete Difficult to synthesize	Required Comprehensive Conducive to systematic review

There are knowledge gaps in construct validity and research methodology between preclinical models and the human condition of sepsis

Another factor affecting the translational potential of preclinical sepsis research is that findings are mostly derived from single investigator/laboratory studies with a bias towards publishing positive results, and concerns with reproducibility and methodological rigour are frequently noted (i.e. lack of sample size calculation, randomization, blinding) [8, 9]. Although the ARRIVE guidelines were introduced as an effort to improve transparency and consistency in preclinical research [10], they have not been consistently adopted. In preclinical sepsis research, significant variations in experimental practice and animal housing/husbandry exist [11], and reporting of experimental methods remains incomplete [8, 9, 12]. Consequently, synthesis and interpretation of results becomes difficult—as has been described in preclinical systematic reviews of sepsis models [13, 14].

Multicentre preclinical studies serve as a promising new strategy for overcoming many of the deficiencies related to methodology and bias. These studies are defined as cooperative research formally conducted in two or more research laboratories with shared protocols and analyses [15]. Although this concept is relatively new in the preclinical environment, multicentre studies have been accepted for decades as the gold standard in clinical research [16]. Well-conducted multicentre preclinical studies maximize transparency, improve reproducibility, enhance internal and external validity (generalizability). Preclinical multicentre studies may increase the efficiency of “bench-to-bedside” translation by identifying replicable and robust findings that future development should focus on [17–19]. Indeed, high-profile multicentre preclinical studies in other fields have provided strong evidence and rationale for either continued or aborted investigation of novel therapeutics being considered for early phase clinical trials [17, 20].

## National Preclinical Sepsis Platform (NPSP): building on Canadian and international strengths

Recent consensus papers by the Wiggers-Bernard Group of preclinical sepsis investigators have outlined several essential domains that should be considered the Minimum Quality Thresholds in Preclinical Sepsis Studies (MQTiPSS) [21]. The panel of international experts established high-level recommendations about model design, methodological practices, and commitment to ethical standards. The knowledge summarized in the MQTiPSS document serves as a valuable roadmap for standardization and design of future preclinical studies, and will require detailed expansion and consideration by research groups around the world.

In Canada, MD, PhD, and allied health scientists working within the Canadian Critical Care Translational Biology Group (CCCTBG, www.ccctg.ca/CCCTBG) have led efforts to study many diseases of critical illness using preclinical approaches. The CCCTBG has sponsored successful sepsis research programmes including rapid diagnostics [22], first-in-human trials for novel therapeutics [23], and mechanisms of disease [24]. The CCCTBG has a longstanding commitment to supporting early career investigators and providing a liaison with clinical researchers in the Canadian Critical Care Trials Group (CCCTG).

In order to incorporate the recommendations of the Wiggers-Bernard Group within the Canadian context, while recognizing the need for high-quality preclinical sepsis research with increased translational impact, we established the National Preclinical Sepsis Platform (NPSP). The NPSP is a collaborative network of Canadian sepsis investigators and stakeholders, intended as a *paradigm shift* for preclinical sepsis research. Our overall goals are to:

1. Create a multicentre infrastructure to rigorously evaluate the pathophysiology of host response and biological heterogeneity of sepsis in a controlled preclinical environment.
2. Adopt an integrated knowledge translation approach (iKT, see next section) to identify shared research goals for preclinical sepsis research that are clinically relevant and patient-centred.
3. Generate adequately powered, high-quality preclinical data for testing safety and efficacy to accelerate the movement of novel therapeutics for sepsis into early phase clinical testing.

Here we describe our activities to date, and provide a detailed summary of the proceedings from our first in-person NPSP meeting, held on June 9, 2019 at the Prince of Wales Hotel (Niagara-on-the-Lake, Ontario, Canada). This meeting was funded by the Ontario Research Fund, the CCCTBG, as well as a Planning and Dissemination Grant from the Canadian Institutes of Health Research (Government of Canada). We believe this framework could be adopted by other groups seeking to strengthen collaborative preclinical critical care research.

## Integrated knowledge translation (iKT) and patient engagement in preclinical research

**Fig. 1:**
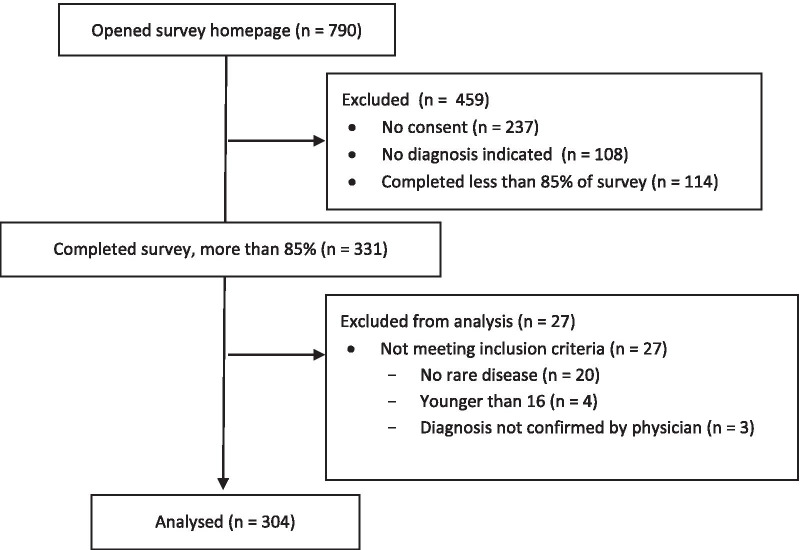
An integrated knowledge translation (iKT) approach to preclinical research, as described for the National Preclinical Sepsis Platform

iKT is an approach to improve the conduct of research by “involving knowledge users as equal partners alongside researchers [25–27]”. iKT is widely applied in clinical research, yet has largely been overlooked in the preclinical research environment. An iKT approach for preclinical sepsis research could engage clinical researchers to refine experimental rationale, identify clinically relevant outcomes, and help select novel therapeutics for future testing. iKT also emphasizes the collaborative role of patients and their caregivers as centrally important and contributing members of the research team (as opposed to clinical research participants). iKT in preclinical sepsis research could ensure alignment with the priorities of sepsis patients and their caregivers, and public-identified areas of interest or concern. By meaningfully engaging with these stakeholders early and throughout the research process, the results of preclinical research may be more likely to be translated into clinical practice. A conceptualized schema is found in Fig. 1.

Within the iKT framework for the NPSP, we note that while patient engagement in clinical research has gained significant momentum [28], the potential impacts and benefits of patient engagement in preclinical research (and sepsis specifically) have not been well-explored. Patient engagement strives to co-create research “with” or “by” patients, rather than “about” or “for” them, and may lead to wider dissemination of research findings, better public education of the value of research, and increased public support and funding for research endeavours [29, 30]. Moreover, patient engagement in preclinical research should be viewed as inherently positive, and aligned with the values of respect, inclusivity, and cooperation between scientists and the public, with a goal of preserving and enhancing the public trust in research. The NPSP engaged a patient partner for our first meeting and will be exploring how to strengthen this partnership as the platform is developed.

## Pre-meeting activities

### Environmental scan

Founding laboratories of the NPSP were first identified through the CCCTBG. Further laboratories were identified through ‘snowball sampling’, where already identified groups suggested additional, potentially interested investigators. After initial coordination via teleconference, and preliminary presentations at national CCCTBG meetings, we secured commitment from a diverse, pan-Canadian group of preclinical sepsis investigators interested in forming the NPSP.

An environmental scan was then performed to understand the current landscape of preclinical sepsis research in Canada. A brief questionnaire was sent to principal investigators and senior highly qualified personnel from each lab (Appendix 1). Eleven laboratories shared preclinical sepsis models and experimental procedures, including granular details of current protocols (e.g. models used, animal species used, monitoring required following disease induction). Given concerns regarding intellectual property, confidentiality was assured, with details only being shared within the group of NPSP collaborators. Outcome measures routinely collected as well as specialized techniques/infrastructure were also requested. Potential barriers for multicentre standardization and harmonization (e.g. animal husbandry) were detailed carefully. These were summarized to compare and contrast current practices across the country, and identify potential areas of strength (e.g. shared models/outcomes) and other areas that will require efforts to harmonize (e.g. differing analgesia protocols required by local animal care services). Individual lab details and the summary were then deposited in a shared online repository to continue building the NPSP collaboration.

### Identification of additional stakeholders

The majority of stakeholders were preclinical sepsis investigators, members of the CCCTBG, and research assistants and trainees directly performing preclinical sepsis experiments. We specifically recruited additional stakeholders from outside this community to participate in the NPSP, including clinical researchers, laboratory animal veterinarians, a patient partner, and clinical research coordinators. We engaged knowledge translation scientists (KMF, JP) to effectively operationalize our efforts. We believe these strategies will strengthen linkages between preclinical and clinical researchers during initial study design, and create formal mechanisms for preclinical results to move rapidly into clinical research. Representation from patient partners was viewed as particularly important to ensure patient and caregiver perspectives were incorporated throughout the NPSP.

## Meeting summary

**Fig. 2:**
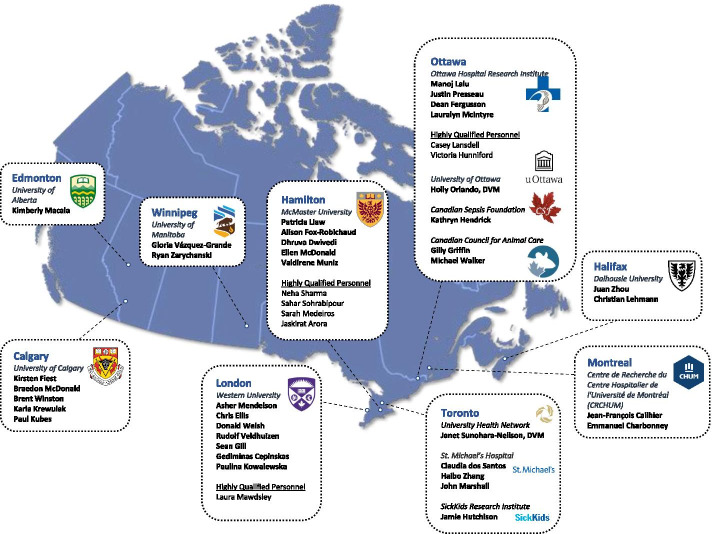
Participants in the National Preclinical Sepsis Platform

The itinerary from the first in-person NPSP meeting can be found in Appendix 2. The goal of the first in-person NPSP meeting was to establish this unique national collaboration and to build consensus on immediate next steps towards implementation of the platform. A participant list for the NPSP meeting can be found in Appendix 3. The total number of participants was 29 of which 10 (35%) were highly qualified personnel (a standard term in Canada used to identify trainees and research personnel [31]). Nine cities from 5 provinces were represented and a total of 10 independent lab groups attended the meeting (Fig. 2). An additional 15 participants were invited but could not attend, giving their regrets. These additional participants contributed to pre- and post-meeting activities and planning.

### Overview of multicentre preclinical studies: results of a systematic review and interview study

A brief overview of the concept and current landscape of multicenter preclinical studies was summarized by an ongoing systematic review and interview study being conducted by several participants (VH, MML, CL) [19]. Based on a systematic search, only 13 multicentre preclinical studies were published from 1985 to 2015, studying diseases in six different areas (none in sepsis). Multicentre studies included in the systematic review were largely performed to confirm preclinical findings prior to testing interventions in humans. As described above, benefits of these studies included increased external validity (i.e. generalizability of findings between laboratories), adequately powered studies, and extensive quality control (e.g. routine, regular oversight). Importantly, interviews of scientists who conducted these studies demonstrated that these were highly collaborative endeavours that benefited from engagement and transparency of all stakeholders involved. Additionally, as noted by the multicentre investigators, these studies were difficult to fund given their novelty and cost, and required greater time and resources than single-centre preclinical research. An important feature of preclinical multicenter projects, and a critical consideration when designing these studies, was the common protocol across participating centres. Multicentre studies were either fully harmonized (identical protocol implementation across laboratories) or triangulated (deliberate between-lab variations in experimental design) depending on the overall objective and scientific hypothesis being tested [19]. Triangulation was particularly beneficial when more than one animal model was tested between centres (i.e. since no single model can recapitulate all features of human pathophysiology).

### Animal ethics and veterinary considerations

This section of the meeting was presented by the director of standards from the Canadian Council for Animal Care (CCAC), the national organization that provides ethical guidance for animal care committees that oversee preclinical research at their respective institutions. CCAC standards (guidelines and policies) are grounded in the 3Rs tenet of replacement, reduction and refinement of animal use. For preclinical protocols with the potential for animals to experience severe suffering (including sepsis models) there is a requirement for more careful ethical review of the protocol, and for more attention to the animals during the course of the study; ultimately, consistent animal care and treatment are essential for the production of robust scientific results. Animal care committees are required to consider the experiences of research animals in relation to the potential benefits to be accrued from the work, and researchers are encouraged to work with the laboratory animal veterinarians at their respective institutions to develop strategies to address these issues. This places an onus on researchers and veterinarians alike to ensure that the outcomes from the studies are reproducible and translatable.

The NPSP provides an opportunity to harmonize protocols between laboratories, and resolve some of the ethical challenges with preclinical sepsis research (for example, defining appropriate endpoints that permit the collection of relevant data, but limit pain and distress for the animals, and determining when analgesia can be provided). While logistical hurdles are inevitable when coordinating between institutions (including the animal care committee reviews), the comprehensive and measured approach of the NPSP is conducive to the reduction and refinement of animal use, thus limiting the wastage of animal lives and ensuring that the data fully contributes to the research record.

### Small group discussion

The details of the environmental scans from each participating laboratory were summarized and presented to the group as a whole. The group then reflected on the similarities and differences between preclinical sepsis models across the country, particularly regarding the severity of sepsis models (lethal vs sub-lethal), co-administration of common therapies (fluids, antibiotics), and duration of study. Subsequently, participants were separated into four small groups. Each group rotated through four discussion topics: (1) the objectives of the NPSP, (2) the sepsis models that the NPSP should pursue, (3) methodology of NPSP experiments, and (4) outcomes that should be studied. This was followed with full-group discussion to summarize small group discussions. Conversations were facilitated by meeting organizers at each table and transcribed in real-time by trainee volunteers.

#### NPSP objectives

Two broad categories of objectives for the NPSP were identified: (a) understand basic mechanisms of sepsis pathophysiology, and (b) assess novel therapeutics for sepsis [32]. Within the therapeutic category, opportunities also exist to study the effect of multiple co-interventions and refinement of conventional therapies. Given how the themes are inter-related, cultivating these two objectives in parallel was viewed as a positive strategy. In addition, the biological variability described by pathophysiology studies can identify sepsis subclasses with common biological features; this data can be used for prognostic or predictive enrichment in clinical trial design [33] or with further testing of novel therapeutics in the preclinical setting. Participants noted that the NPSP can serve as a quality assurance checkpoint for research before considering clinical testing in humans. This may require single-centre research to be presented to the NPSP and replicated across the platform in a multicentre fashion.

#### Sepsis models

Participants discussed various models commonly used by preclinical sepsis scientists in Canada; universally these were rat and murine models, with merits and drawbacks for both species. Advantages for murine models include shorter reproductive cycles, economical housing/maintenance costs, and availability of genetically modified strains. Conversely, the use of invasive haemodynamic monitors (e.g. blood pressure) and mechanical ventilation is possible in rats, but more technically challenging in mice. The integration of predisposing conditions (e.g. diabetes, age, biological sex) into the sepsis models was viewed as particularly relevant for clinical translation. These co-morbid animals would reflect patients at increased risk for developing sepsis and for poor sepsis outcomes.

In terms of model specifics, participants recognized that a “gold standard” model for sepsis does not exist, and all models have benefits and drawbacks; the group concurred with MQTiPSS that endotoxemia was not representative of clinical sepsis. Discrepancy was noted between preclinical sepsis models where the time of infectious insult is known, and clinical sepsis where presentation to the emergency department can occur at variable time points in the disease process.

Overall, participants agreed that minimizing surgical variability by selecting less challenging and more technically simple sepsis models was a priority for the platform. The value of investigating models outside of traditional abdominal Gram-negative sepsis was recognized. Participants further agreed that efforts should be made to develop/include at least two sepsis models for the platform. Finally, although the participants noted the existence of robust long-term (e.g. up to 28 days) preclinical models of sepsis [34, 35], there was agreement that this type of model would require intensive personnel support and incur much higher costs. Given that acute sepsis models (< 24 h) are much more common, this leads to under-representation of the late phase of the disease in preclinical experimentation, despite its significant contribution to human sepsis deaths [36].

The “PIRO” model (predisposition, insult, response, organ dysfunction) was accepted as an overall experimental framework for the platform. PIRO was first introduced as a scoring system for acute illness in clinical settings [37]. By applying PIRO to preclinical sepsis research, we link our experimental design with clinical and population health researchers using a common language. This framework also provides a consistent structure and reproducible design for future studies, including the potential evaluation of novel therapeutic interventions.

#### Methodology to increase reproducibility and reduce bias

The key tenets of improving internal validity/methodological rigour were explained (e.g. blinding, randomization, sample size calculation), and participants discussed how to incorporate them into the NPSP. The concept of protocol harmonization was discussed; overall, participants agreed that full harmonization of basic protocols would initially yield greater advantages versus a ‘triangulation’ approach (where a common intervention is tested but models, protocols, and outcomes remain unique between labs). Harmonization would also ensure that processes that are implicit in each participating laboratory are made explicit, and that previously unaccounted variation could be  considered. While some heterogeneity between labs may be addressed with technical training sessions, other heterogeneity between centres (e.g. housing conditions, commensal flora) would still remain. These variations were thought to be potentially beneficial to assess generalizability of findings to the clinical setting where heterogeneity is common. Standardization could be facilitated for centres with varying levels of technical expertise using detailed and descriptive standard operating procedures; video training modules and in-person demonstrations were both mentioned. Participants recognize that even with efforts to achieve total standardization, variability will persist due to the inherent biological heterogeneity of sepsis. By controlling for variability as much as possible, however, the platform will help determine the impact of this biological heterogeneity on sepsis outcomes (e.g. by stratifying according to confounders that cannot be controlled).

Validation of models and quality assurance across multiple laboratories was recognized as essential to ensure reproducibility and generalizability of results. The participants discussed potential methods for this to be achieved, including use of a preclinical scoring system to assess disease severity that is standardized and clinically relevant [38]. In order to address issues of bias that are very common for preclinical laboratory studies [12], methods to increase internal validity of studies were discussed. For instance, selection bias can be minimized with a priori inclusion/exclusion criteria that ascertain whether the animals have achieved sepsis (e.g. scoring system, biomarkers, bacterial load). In addition, sample sizes should be calculated a priori based on current literature and data from pilot studies to adequately power NPSP studies to detect differences between experimental groups. Finaly, an independent, arms-length committee for oversight and quality assurance was also suggested, similar to a Drug and Safety Monitoring Committee in clinical trials. This committee would review proposed protocols and interventions, and provide both scientific and quality control advice for NPSP investigators.

Participants agreed that sharing of biological specimens for centralized analysis would be optimal, but that it would require many additional logistical and cost considerations. Coordinating animal ethics approval across multiple centres was seen as a potential barrier for many investigators but the veterinarian stakeholders believed it could be addressed with appropriate communication and coordination between centre veterinarians. Participants noted that study protocols should be transparent, registered (e.g. www.preclinicaltrials.eu) [39], and potentially published ahead of time. The value of publishing negative results from the platform was also recognized, as these could inform future study designs.

#### Outcomes for evaluation

It was agreed by participants that the NPSP should evaluate clinically relevant outcomes, but also capture the fundamental pathophysiology of sepsis (i.e. biological outcomes). Efforts should be made to align the NPSP with outcomes that matter to patient partners and caregivers; this relates particularly with long-term outcomes for mobility and cognitive function. Ethical considerations were raised for mortality outcomes in animal models of sepsis [32] and the need to identify appropriate “mortality surrogates” and humane endpoints that can be used to correlate with meaningful clinical outcomes [40]. Biological outcomes should address the scope of sepsis including immunology, coagulation, microcirculation, and cellular metabolism. NPSP participants agreed that there should be a basic panel of outcomes measured across all sites, as well as site-specific outcomes based on nationally recognized expertise. Multiple organ systems should be evaluated with outcomes that can best align with clinical research; functional outcomes may be challenging to evaluate in the preclinical setting given that they often represent complex processes (e.g. cognition, mobility). In addition, given the paucity of safety data in published preclinical sepsis studies, it was agreed that studies for novel therapeutics should include a priori defined safety outcomes in addition to efficacy data.

### Keynote address: Dr. Marcin Osuchowski, MQTiPSS, Wiggers-Bernard Group

Dr. Osuchowski connected via videoconference to the NPSP meeting, to deliver a keynote address on the design and creation of the MQTiPSS recommendations. Insights were shared regarding logistical considerations for organizing a large team of preclinical researchers, as well as the dialogue and consensus approaches that were adopted for the project.

Dr. Osuchowski reported the 10th Wiggers-Bernard Conference Initiative on Pre-clinical Modeling in Sepsis (www.wiggers-bernard.org) has launched an international multicentre preclinical sepsis trial; the details and logistics were discussed at the 2019 Wiggers-Bernard Meeting (Chania, Crete, Greece). Dr. Osuchowski commended the NPSP participants for undertaking a similar initiative in Canada, and all participants expressed a desire for ongoing communication and shared discussions as these projects continue to evolve.

### Patient engagement at the NPSP

Although preclinical researchers and patient partners shared a common goal and purpose (i.e. improving treatment for sepsis patients), NPSP participants felt that it was unclear how patient partner skills and lived experiences could be successfully integrated into this laboratory-focused endeavour. Common language and frame of reference was lacking, which prevented a substantive discussion about preclinical research collaboration. This was identified as a key barrier despite the positive attitude and efforts by participants and our patient partner. Accordingly, we have undertaken a critical examination of this issue with a scoping review for effective strategies to achieve successful patient-preclinical engagement. In addition, it was felt that having multiple patient partners engaged in future NPSP meetings would improve representation and better reflect a diversity of patient and caregiver perspectives.

## Future directions

The NPSP brings together a diverse set of stakeholders from across Canada and represents an innovative and exciting resource for preclinical sepsis research. Through foundational discussions at our first in-person meeting, participants were able to achieve consensus on the overall objective (i.e. to undertake multicentre preclinical studies evaluating the pathophysiology of sepsis), and identified a number of specific issues that require more planning (e.g. protocol harmonization, research questions).

Further discussions are needed to clarify data management procedures and ethics approval between centres, which will be a priority for the upcoming success of this platform. Moreover, participants noted that external funding opportunities will be needed to ensure the sustainability of the platform. Participants will be encouraged to develop lines of research that can leverage the NPSP infrastructure while also building on the unique strengths and expertise of individual investigators.

There are short-term and medium-term objectives for the NPSP that we plan to accomplish. In support of these endeavours, Sepsis Canada (an interdisciplinary network dedicated to sepsis research) was recently funded by the Canadian Institutes of Health Research. This network will invest in infrastructure required for NPSP operations. Since the meeting, we have continued to form consensus regarding details of the animal models and outcomes for evaluation. Moreover, scientific hypotheses within the PIRO framework (e.g. effect of predisposing conditions on sepsis outcomes) are being elaborated with preclinical systematic reviews to assist with study design. Finally, we have started to plan the first series of pilot experiments that will serve to demonstrate the feasibility of this platform, and allow us to refine the logistics needed for coordinating a multicentre preclinical platform.

In conclusion, we hope that details outlined by these proceedings will serve as a template and inspiration for other investigators who are seeking to establish similar collaborative projects. The creation of high-quality preclinical research should be viewed as a priority for the sepsis research community, and multicentre preclinical research can help maximize the translational impact of preclinical findings. The success of these endeavours will require dedication and teamwork. We are confident that the NPSP will elucidate new insights into sepsis pathogenesis, and accelerate the development of improved treatment strategies for patients with sepsis.

## Data Availability

Not applicable.

## Appendix 1. Questions for initial environmental scan of preclinical sepsis laboratories

PI and institute:

**Table:**

Variable
*Experimental model*
Name of model
Details of disease induction method
Timing of sacrifice (what is the latest timepoint following disease induction)
Analgesia (if any, type, dose and timing)
Antibiotics (if any, type, dose, and timing)
Fluids (if any, type, dose, and timing)
Physiological monitoring details (e.g. temperature, heart rate, etc., and materials used)
Physiological monitoring frequency (e.g. every hour, specific timepoints)
Method of sacrifice (e.g. pentobarbital overdose)
Outcomes you have used/reported on with this model (provide timing and general method used (e.g. plasma cytokines; 3, 6, 12, 24 h; ELISA)
Humane endpoints (details from animal care)
*Animal*
Species
Background
Wild type or mutant (+ type of variation)
Vendor
Sex
Age
Average # of animals per experiment
# animals per cage
Animal ID (how do you label/ID animals; ear notch/tags/tail markings)
Location of housing (e.g. conventional, biohazard, stepdown)
*Husbandry*
Diet
Light cycle
Health status of rooms
Noise level/traffic of rooms
Bedding type
Water treatment (e.g. acidified)
Type of cage
Enrichment (e.g. cardboard house)
Frequency of cage change
Timing of cage change relative to the intervention
*Animal care settings*
Room temperature
Relative humidity
Air changes per hour
*Publications*
Any publications that highlight your use of this model?
*Additional comments*

## Appendix 2. National Preclinical Sepsis Platform—meeting agenda

**Table:**

Time	Topic	Speaker
10:45–11:00	Registration, Sign-In	
11:00–11:30	Introductions, overview of day, ice-breaker	Asher Mendelson
11:30–11:45	Why should we conduct preclinical multicenter studies?	Manoj Lalu
11:45–12:00	Barriers and facilitators to preclinical multicenter studies	Victoria Hunniford
12:00–12:45	Applying a framework of integrated knowledge translation (iKT) to preclinical research	Kirsten Fiest
12:45–13:15	Animal ethics and collaboration with laboratory animal veterinarians	Gilly Griffin and Janet Sunohara-Neilson
13:15–13:45	Lunch	
13:45–14:45	Review of environmental scan: what is the landscape of Canadian preclinical sepsis research?	Manoj Lalu
14:45–15:30	Keynote Address: MQTiPSS guidelines and beyond: a personal insight on how to improve the quality of sepsis modeling	Marcin Osuchowski
15:30–15:45	Break for refreshments and group photo	
15:45–17:05	Building Consensus on a Canadian Preclinical Sepsis Platform Table 1 Sepsis Models: Which models are feasible and clinically relevant? Table 2 Purpose of NPSP: What studies should the platform undertake (e.g. therapies, biomarkers, pathophysiology)? Table 3 Methodology: What methods/logistics considerations are needed for the platform? Table 4 Outcomes: What outcomes are important for translation to patients?	Facilitator: Alison Fox-Robichaud Everyone will participate in 20 min discussions per table, then rotate Trainees are notetakers
17:05–17:20	Break	
17:20–18:40	Tables report back on group discussions	All
18:40–19:00	Next steps, Canadian Sepsis Network proposal discussion, and closing remarks	Manoj Lalu, Asher Mendelson and Alison Fox-Robichaud
19:30	Networking Dinner: Noble Restaurant, Prince of Wales Hotel	

## Appendix 3. List of participants at the National Preclinical Sepsis Platform meeting. There were 29 people in attendance and 15 gave their regrets but contributed through teleconferences and other communication pre- and post-meeting. *Organizing committee

**Table:**

Name	Primary affiliations
Alison Fox-Robichaud*	Staff Physician, Department of Medicine, Division of Critical Care, Hamilton Health Sciences, Hamilton, Ontario Scientist, Department of Medicine and Thrombosis and Atherosclerosis Research Institute, McMaster University, Hamilton, Ontario
Asher Mendelson*	Staff Physician, Intensive Care Unit, St. Mary’s General Hospital, Kitchener, Ontario Adjunct Scientist, Centre for Critical Illness Research, Lawson Health Research Institute, London, Ontario Ph.D. Candidate, Department of Medical Biophysics, Schulich School of Medicine & Dentistry, University of Western Ontario, London, Ontario
Casey Lansdell*	Research Assistant, Department of Regenerative Medicine, Ottawa Hospital Research Institute, Ottawa, Ontario
Chris Ellis	Scientist, Department of Medical Biophysics, Schulich School of Medicine & Dentistry and Robarts Research Institute, University of Western Ontario, London, Ontario
Christian Lehmann	Scientist, Department of Anesthesia, Pain Management & Perioperative Medicine, Dalhousie University, Halifax, Nova Scotia
Claudia dos Santos	Staff Physician, Department of Medicine, Division of Respirology, St. Michael’s Hospital, Toronto, Ontario Scientist, Keenan Research Centre for Biomedical Science, St. Michael’s Hospital, Toronto, Ontario
Dhruva Dwivedi	Postdoctoral Fellow, Department of Medicine, Division of Hematology and Thromboembolism, McMaster University, Hamilton, Ontario Research Associate, Thrombosis and Atherosclerosis Research Institute, McMaster University, Hamilton, Ontario
Donald Welsh	Scientist, Department of Physiology and Pharmacology, Schulich School of Medicine & Dentistry and Robarts Research Institute, University of Western Ontario, London, Ontario
Ellen McDonald	Research Coordinator, Department of Medicine, McMaster University, Hamilton, Ontario and the Thrombosis and Atherosclerosis Research Institute, Hamilton, Ontario
Gilly Griffin	Director of Standards, Standards Setting and Maintenance unit, Canadian Council on Animal Care, Ottawa, Ontario
Gloria Vázquez-Grande	Critical Care External Clinician Fellow, Intensive Care Unit, Winnipeg Regional Health Authority, Winnipeg, Manitoba Ph.D. Candidate, Department of Medical Microbiology and Infectious Diseases, University of Manitoba, Winnipeg, Manitoba
Jamie Hutchison	Staff Physician, Department of Critical Care Medicine, The Hospital for Sick Children, Toronto, Ontario Senior Scientist, Neuroscience and Mental Health Research Program, SickKids Research Institute, Toronto, Ontario
Janet Sunohara-Neilson	Clinical Veterinarian, University Health Network, Toronto, Ontario
Jean-François Cailhier	Staff Physician, Department of Medicine, Institut du cancer de Montréal, Montreal, Quebec Senior Scientist, Department of Medicine, Centre de Recherche du Centre Hospitalier de l’Université de Montréal (CRCHUM), Montreal, Quebec
Juan Zhou	Scientist, Department of Anesthesia, Pain Management & Perioperative Medicine, Dalhousie University, Halifax, Nova Scotia
Kathryn Hendrick	Patient Partner Advocate for improved sepsis management, Ottawa, Ontario Volunteer Communications Director, Global Sepsis Alliance, Canada Sector, Toronto, Ontario Canada Board Member, Canadian Sepsis Foundation, Markham, Ontario
Kimberly Macala	Staff Physician, Division of Critical Care, Royal Alexandra Hospital, Edmonton, Alberta Scientist, Department of Critical Care Medicine, University of Alberta, Edmonton, Alberta
Kirsten Fiest	Scientist, Department of Critical Care Medicine, Community Health Sciences and Psychiatry, University of Calgary, Calgary, Alberta
Laura Mawdsley	M.Sc. Candidate, Department of Medical Biophysics, Schulich School of Medicine & Dentistry, University of Western Ontario, London, Ontario
Manoj Lalu*	Staff Physician, Department of Anesthesiology and Pain Medicine, The Ottawa Hospital, Ottawa, Ontario Associate Scientist, Regenerative Medicine and Clinical Epidemiology Programs, The Ottawa Hospital Research Institute, Ottawa, Ontario
Michael Walker	Senior Research Analyst, Canadian Council on Animal Care, Ottawa, Ontario
Neha Sharma	Ph.D. Candidate, Department of Medical Sciences, McMaster University, Hamilton, Ontario and the Thrombosis and Atherosclerosis Research Institute, Hamilton, Ontario
Patricia Liaw*	Scientist, Department of Medicine, McMaster University, Hamilton, Ontario and the Thrombosis and Atherosclerosis Research Institute, Hamilton, Ontario
Rudolf Veldhuizen	Scientist, Critical Illness Research Program, Lawson Health Research Institute and Departments of Medicine and Physiology & Pharmacology, Schulich School of Medicine & Dentistry, University of Western Ontario, London, Ontario
Sahar Sohrabipour	M.Sc. Candidate, Department of Medical Sciences, McMaster University, Hamilton, Ontario and the Thrombosis and Atherosclerosis Research Institute, Hamilton, Ontario
Sarah Medeiros	Ph.D. Candidate, Department of Medical Sciences, McMaster University, Hamilton, Ontario and the Thrombosis and Atherosclerosis Research Institute, Hamilton, Ontario
Sean E Gill	Scientist, Critical Illness Research Program, Lawson Health Research Institute and Departments of Medicine and Physiology & Pharmacology, Schulich School of Medicine & Dentistry, University of Western Ontario, London, Ontario
Valdirene S Muniz	Postdoctoral Fellow, Department of Medical Sciences, McMaster University, Hamilton, Ontario and the Thrombosis and Atherosclerosis Research Institute, Hamilton, Ontario
Victoria Hunniford	M.Sc. Candidate, Telfer School of Management, University of Ottawa, Ottawa, Ontario
*Regrets:*	
Braedon McDonald	Staff Physician, Foothills Medical Centre, Rockyview General Hospital and South Health Campus, Calgary, Alberta Scientist, Snyder Institute for Chronic Diseases and International Microbiome Centre, Cumming School of Medicine, University of Calgary, Calgary, Alberta
Brent Winston	Scientist, Immunology Research Group and Airway Inflammation Research Group, Snyder Institute for Chronic Diseases, Cumming School of Medicine, University of Calgary, Calgary, Alberta
Dean Fergusson	Director and Senior Scientist, Clinical Epidemiology Program, Ottawa Hospital Research Institute, Ottawa, Ontario
Emmanuel Charbonney	Staff Physician, Intensive Care Unit, Département de Médecine, Université de Montréal, Montreal, Quebec Scientist, Centre de Recherche du Centre Hospitalier de l’Université de Montréal, Montreal, Quebec
Gediminas Cepinskas	Director and Scientist, Centre for Critical Illness Research, Lawson Health Research Institute and Department of Medical Biophysics, Schulich School of Medicine & Dentistry, University of Western Ontario, London, Ontario
Haibo Zhang	Staff Physician, Interdepartmental Division of Critical Care Medicine; Departments of Anesthesia and Physiology, University of Toronto, Ontario Scientist, Keenan Research Centre for Biomedical Science, Department of Physiology, St. Michael’s Hospital, Toronto, Ontario
Holly Orlando	Director and Veterinarian, Animal Care and Veterinary Service, University of Ottawa, Ottawa, Ontario
John Marshall	Staff Physician, Departments of Surgery and Critical Care Medicine, St. Michael’s Hospital, Toronto, Ontario Co-Director and Senior Scientist, Critical Illness and Injury Research Centre, Keenan Research Centre for Biomedical Science, St. Michael’s Hospital, Toronto, Ontario
Justin Presseau	Scientist, Clinical Epidemiology Program, Ottawa Hospital Research Institute, Ottawa, Ontario
Karla Krewulak	Senior Research Associate, Department of Critical Care Medicine, University of Calgary, Calgary, Alberta
Lauralyn McIntyre*	Staff Physician, Department of Medicine, Division of Critical Care, The Ottawa Hospital, Ottawa, Ontario Senior Scientist, The Ottawa Hospital Research Institute, Ottawa, Ontario
Paul Kubes	Scientist, Department of Physiology and Pharmacology, Immunology Research Group and Snyder Institute for Chronic Diseases, Cumming School of Medicine, University of Calgary, Calgary, Alberta
Paulina Kowalewska	Postdoctoral Fellow, Robarts Research Institute, University of Western Ontario, London, Ontario
Ryan Zarychanski	Staff Physician, Department of Internal Medicine, University of Manitoba, Winnipeg, Manitoba Senior Scientist, Department of Medicine, Sections of Critical Care and of Hematology/Medical Oncology, Research Institute of Oncology and Hematology, University of Manitoba, Winnipeg, Manitoba

## Abbreviations

CCAC: Canadian Council for Animal Care
CCCTBG: Canadian Critical Care Translational Biology Group
iKT: Integrated knowledge translation
MQTiPSS: Minimum quality thresholds in preclinical sepsis studies
NPSP: National Preclinical Sepsis Platform
PIRO: Predisposition, insult, response, organ dysfunction
